# Supplementary material for: Anti-Retroviral Protease Inhibitors Regulate Human Papillomavirus 16 Infection of Primary Oral and Cervical Epithelium
Source: Cancers (Basel). 2020 Sep 18;12(9):2664. doi: 10.3390/cancers12092664 (PMC7563395; doi:10.3390/cancers12092664)
Supplement: Supplementary file 1 [file cancers-12-02664-s001.pdf]

Supplementary Materials: Anti-Retroviral Protease Inhibitors Regulate Human Papillomavirus 16 Infection of Primary Oral and Cervical Epithelium

Samina Alam, Sreejata Chatterjee, Sa Do Kang, Janice Milici, Jennifer Biryukov, Han Chen and Craig Meyers

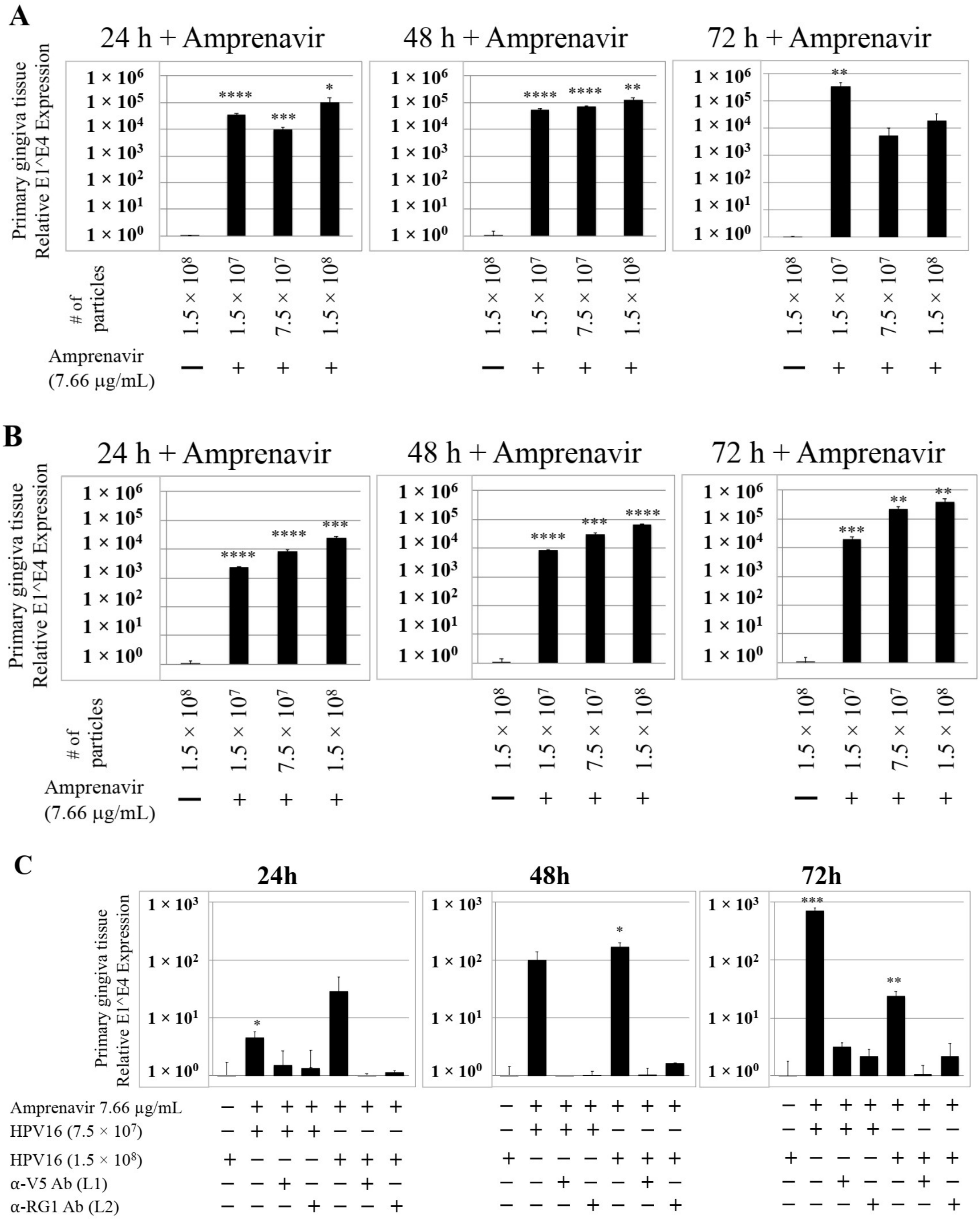

D

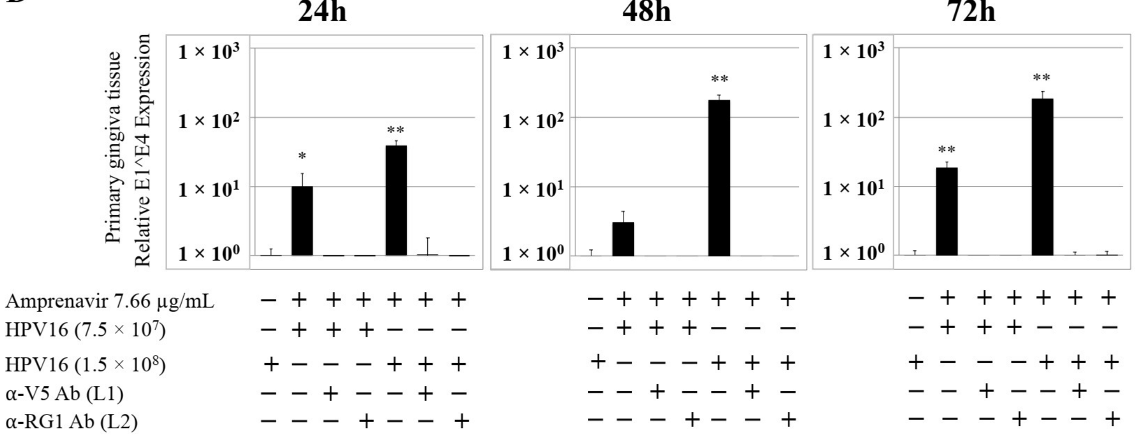
24h
48h
72h
Primary gingiva tissue
Relative E1^E4 Expression
1 × 10³
1 × 10²
1 × 10¹
1 × 10⁰
*
**
Amprenavir 7.66 µg/mL
HPV16 (7.5 × 10⁷)
HPV16 (1.5 × 10⁸)
α-V5 Ab (L1)
α-RG1 Ab (L2)
− + + + + + +
− + + + − − −
+ − − − + + +
− − + − − + −
− − − + − − +

E

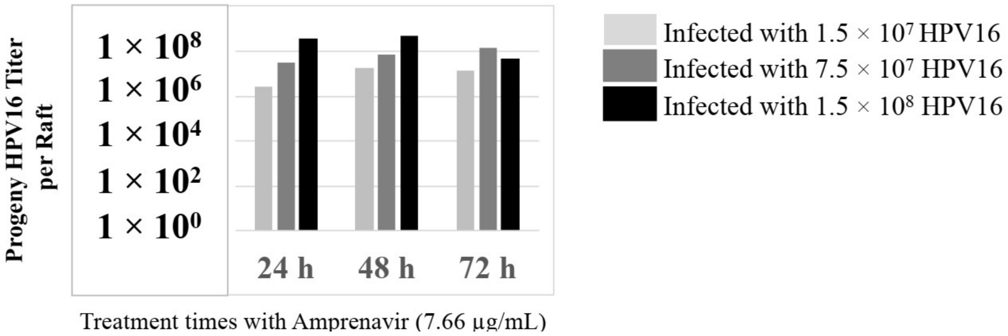
Progeny HPV16 Titer per Raft
1 × 10⁸
1 × 10⁶
1 × 10⁴
1 × 10²
1 × 10⁰
24 h
48 h
72 h
Treatment times with Amprenavir (7.66 µg/mL)
Infected with 1.5 × 10⁷ HPV16
Infected with 7.5 × 10⁷ HPV16
Infected with 1.5 × 10⁸ HPV16

F

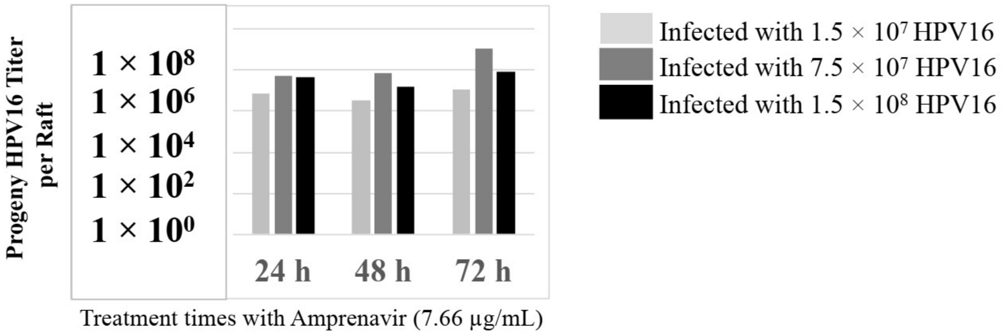
Progeny HPV16 Titer per Raft
1 × 10⁸
1 × 10⁶
1 × 10⁴
1 × 10²
1 × 10⁰
24 h
48 h
72 h
Treatment times with Amprenavir (7.66 µg/mL)
Infected with 1.5 × 10⁷ HPV16
Infected with 7.5 × 10⁷ HPV16
Infected with 1.5 × 10⁸ HPV16

G

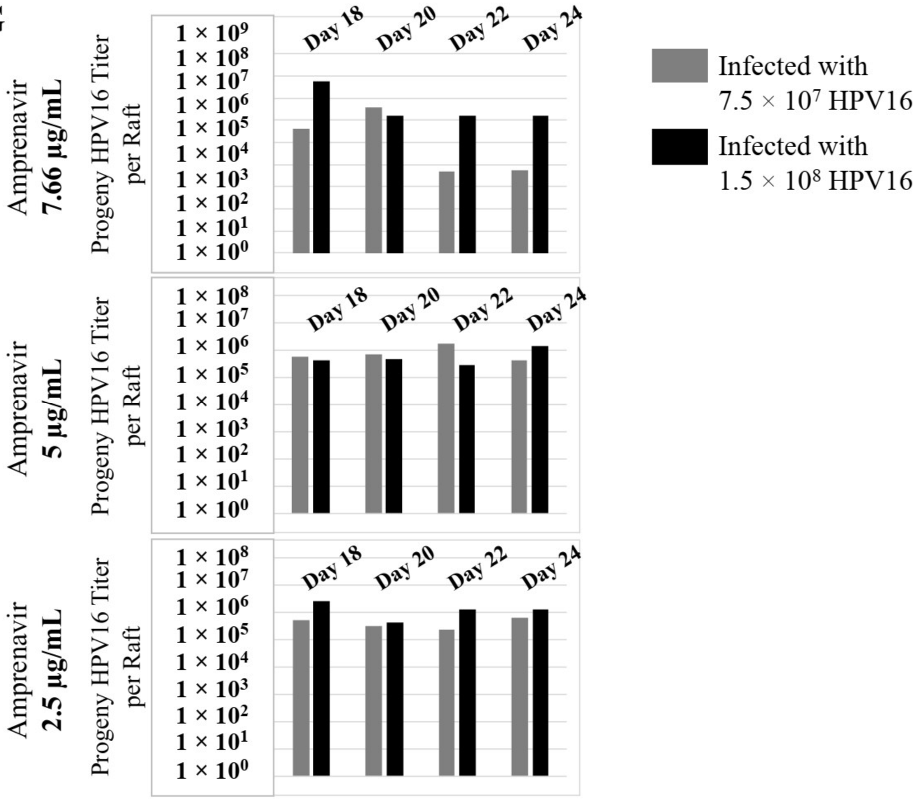

H

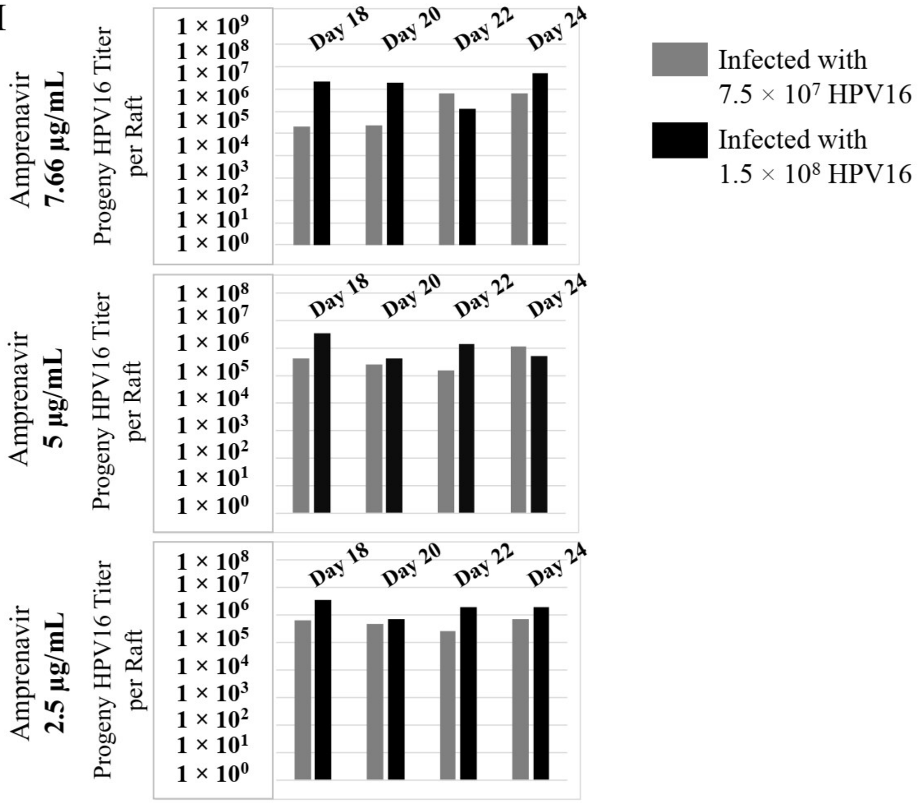

Figure S1. Amprenavir (7.66 μg/mL) Treatment Sensitizes Primary Gingiva Tissue to HPV16 Infection. (**A,B**) Comparative expression of HPV16 E1^E4 transcripts in Amprenavir treated tissues compared with virus infected tissues not drug treated. (**C,D**) Inhibition of virus infection of Amprenavir treated tissues using HPV16 pre-incubated with α-V5 and α-RG1. Data was analyzed as mean ± SD. *p*-values were calculated using two-tailed Student's *t*-tests (Graph Pad Prism). Quantitative data are presented as mean ± standard deviation. Significance was based on pairwise

Student's *t*-test. Comparisons are indicated as $0.01 < p < 0.05$ by *; $0.001 < p < 0.01$ by **; $0.0001 < p < 0.001$ by ***; and $p < 0.0001$ by ****. (**E,F**) *Progeny*-HPV16 virus stock titers isolated from raft tissues infected with three virus doses indicated in Light Grey bars: 1.5×10^7 HPV16 virions; Grey bars: 7.5×10^7 HPV16 virions; Black bars: 1.5×10^8 HPV16 virions. (**G,H**) Extended culturing of raft tissues (day 18–24) modulates *prog*-HPV16 titers in an Amprenavir concentration dependent manner.

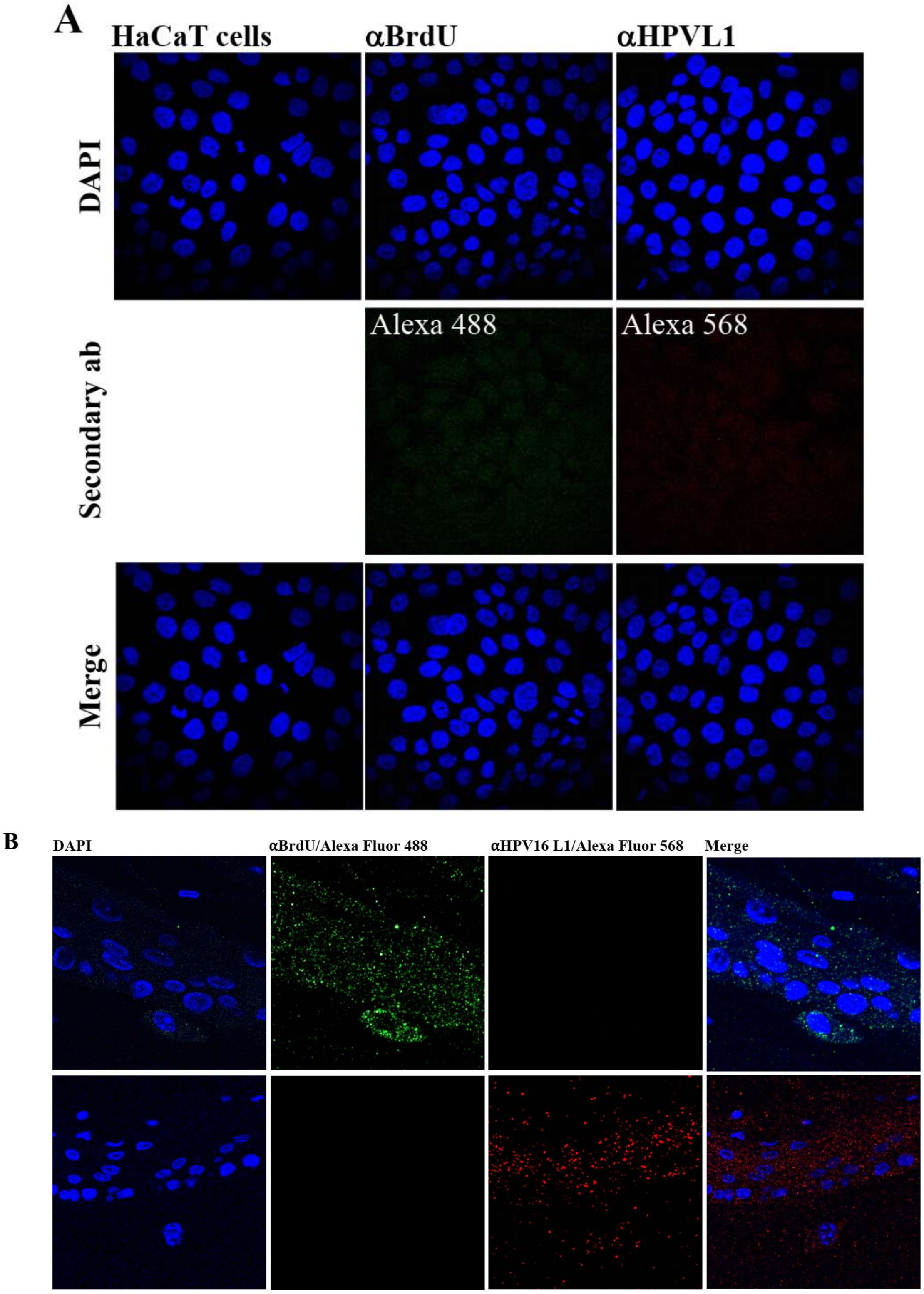

Figure S2. Control staining for confocal imaging. (**A**) HaCaT cells alone do not bind non-specifically to α-BrdU and α-L1 antibodies. (**B**) "Bleed-through" crossover controls for confocal immunofluorescence of gingiva raft tissues treated with Amprenavir and infected with HPV16-BrdU.

Primary gingiva derived raft tissues treated with Amprenavir (7.66 μg/mL) and layered with *P*-HPV16-BrdU for 48h were harvested, sectioned and slides were stained individually for (top panel) BrdU-labeled genomes (detected with Alexa Fluor 488) and (bottom panel) HPV16 L1 (detected with Alexa Fluor 568). Crosstalk fluorescence in the red and green channels was not observed.

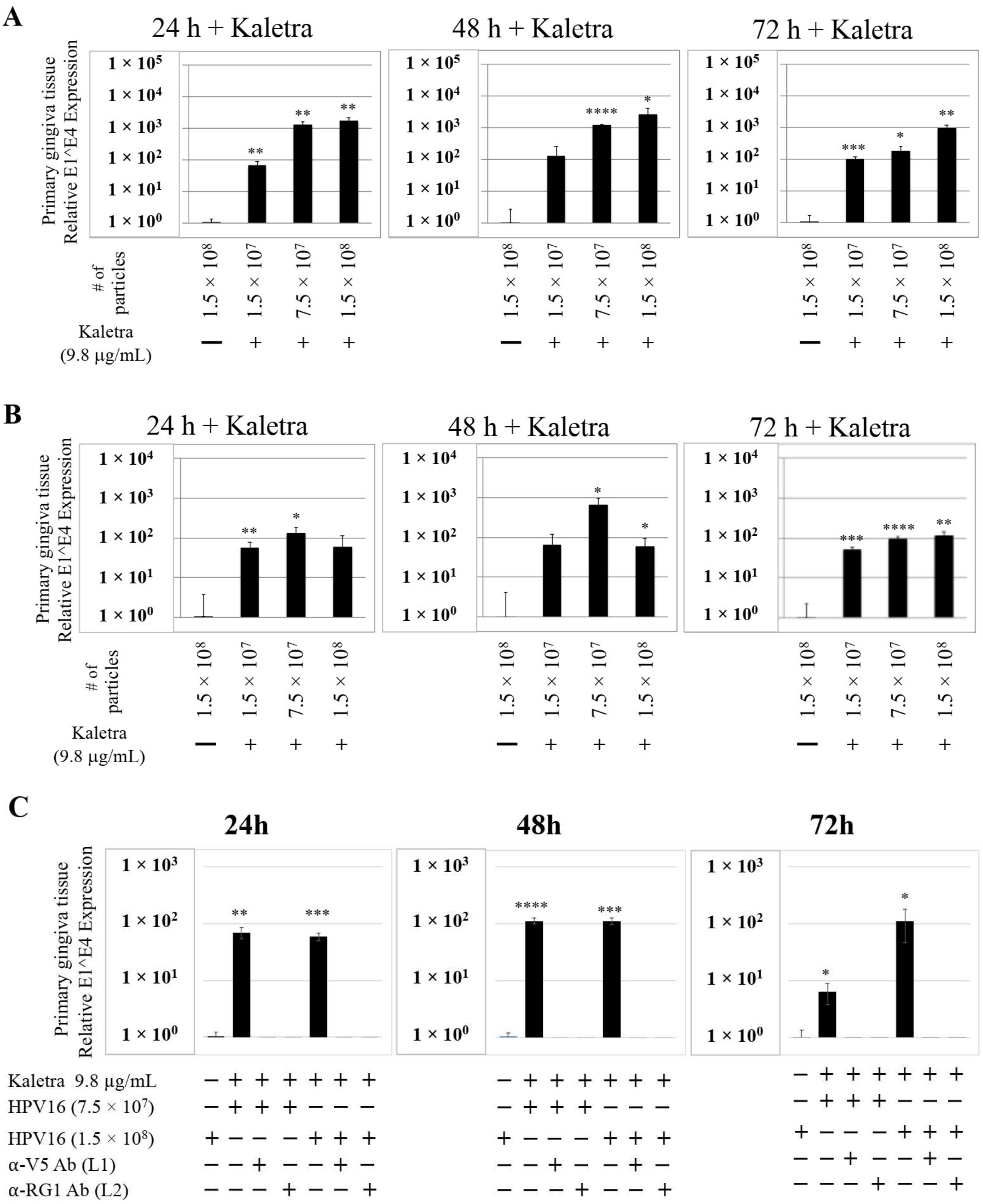

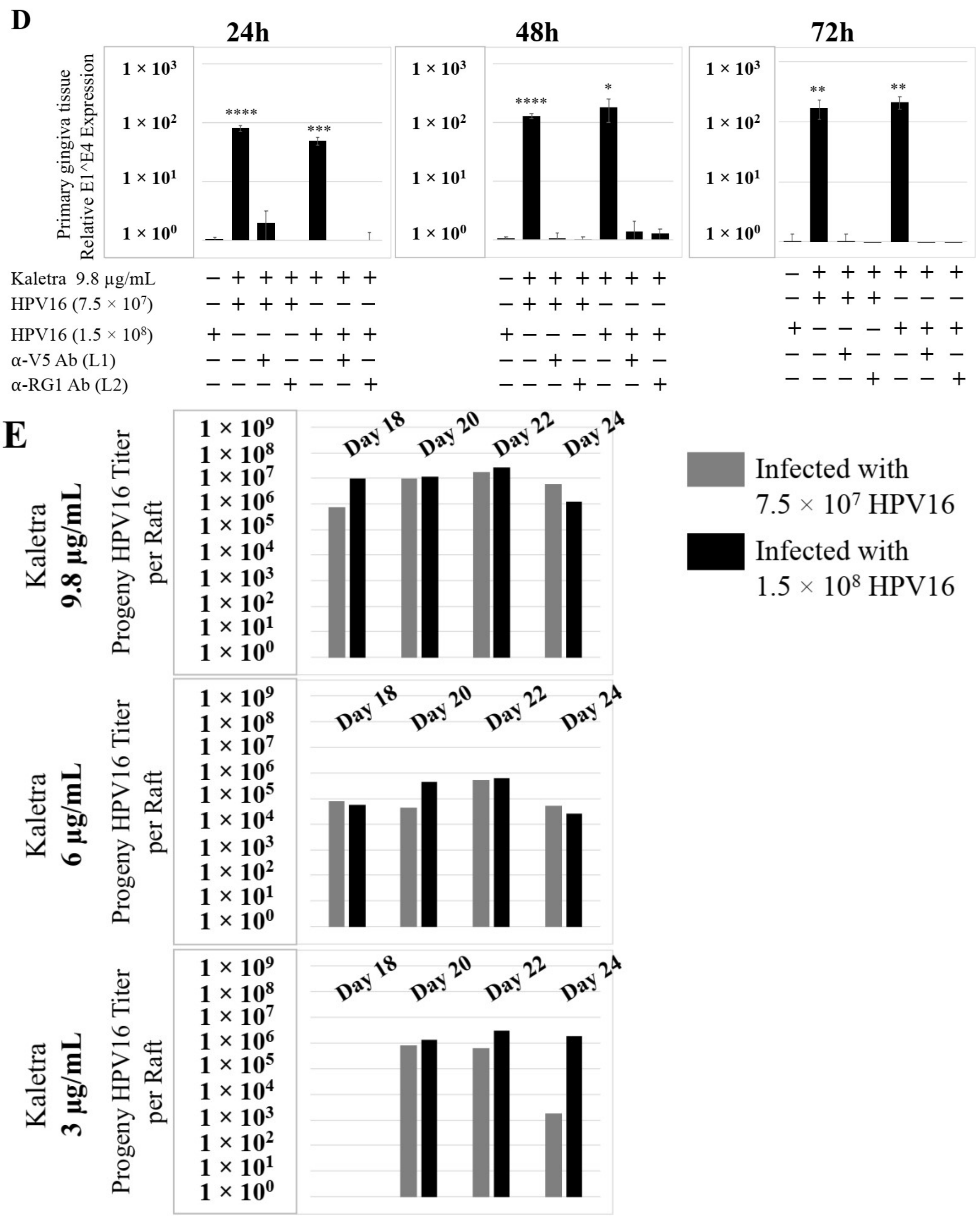
D
24h
48h
72h
Primary gingiva tissue
Relative E1^E4 Expression
1 × 10³
1 × 10²
1 × 10¹
1 × 10⁰
Kaletra 9.8 µg/mL
HPV16 (7.5 × 10⁷)
HPV16 (1.5 × 10⁸)
α-V5 Ab (L1)
α-RG1 Ab (L2)
E
Kaletra 9.8 µg/mL
Kaletra 6 µg/mL
Kaletra 3 µg/mL
Progeny HPV16 Titer per Raft
1 × 10⁹
1 × 10⁸
1 × 10⁷
1 × 10⁶
1 × 10⁵
1 × 10⁴
1 × 10³
1 × 10²
1 × 10¹
1 × 10⁰
Day 18
Day 20
Day 22
Day 24
Infected with 7.5 × 10⁷ HPV16
Infected with 1.5 × 10⁸ HPV16

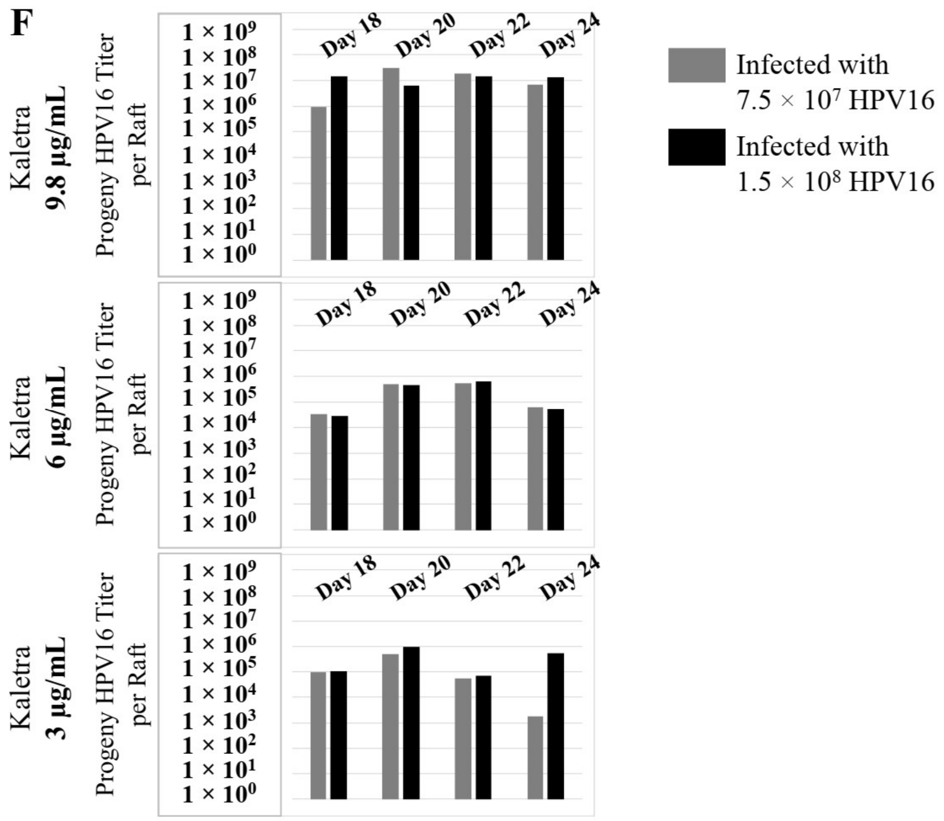

Figure S3. Kaletra (9.8 µg/mL) Treatment Sensitizes Primary Gingiva Tissue to HPV16 Infection. Note: Each panel indicates an individual experiment. (**A,B**) Comparative expression of HPV16 E1^E4 transcripts in Kaletra treated tissues compared with virus infected tissues not drug treated. (**C,D**) Inhibition of virus infection of Kaletra treated tissues using HPV16 pre-incubated with α-V5 and α-RG1. Data was analyzed as mean ± SD. *p*-values were calculated using two-tailed Student's *t*-tests. Significance was based on pairwise Student's *t*-test. Comparisons are indicated as $0.01 < p < 0.05$ by *; $0.001 < p < 0.01$ by **; $0.0001 < p < 0.001$ by ***; and $p < 0.0001$ by ****. (**E,F**) Extended culturing of raft tissues (day 18–24) modulates *prog*-HPV16 titers in a Kaletra concentration dependent manner.

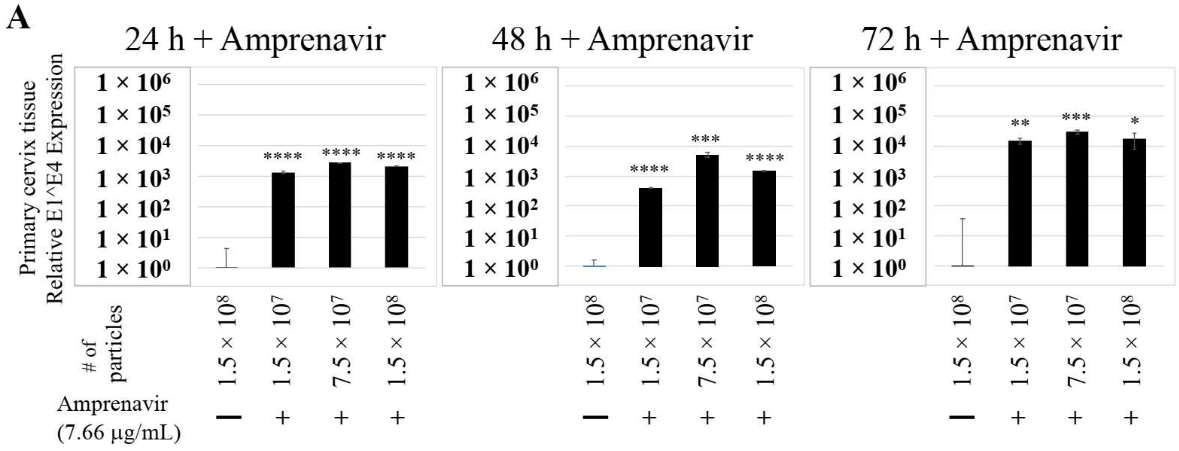

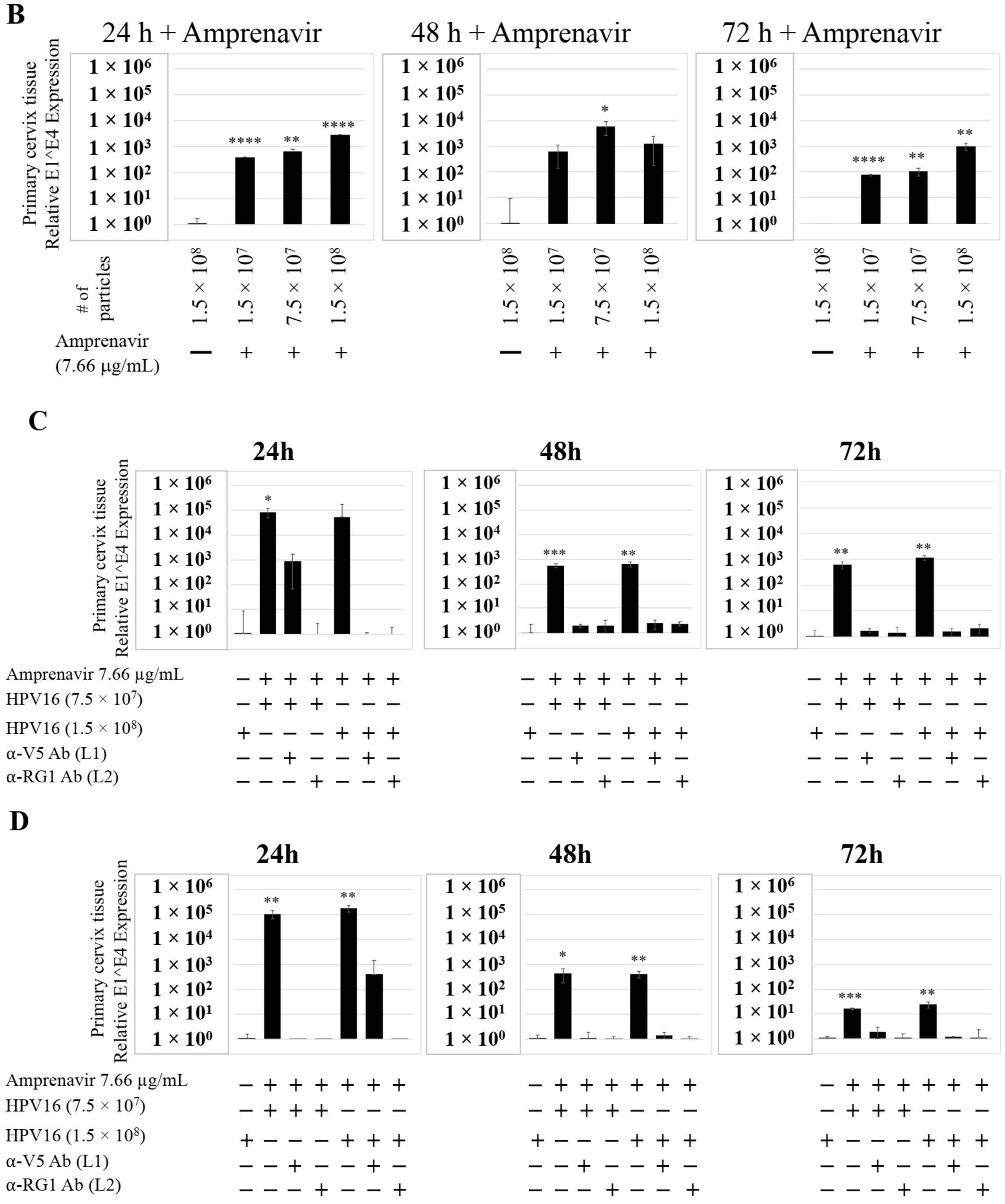
B
24 h + Amprenavir
48 h + Amprenavir
72 h + Amprenavir
Primary cervix tissue Relative E1^E4 Expression
1 × 10⁶
1 × 10⁵
1 × 10⁴
1 × 10³
1 × 10²
1 × 10¹
1 × 10⁰
# of particles
1.5 × 10⁸ 1.5 × 10⁷ 7.5 × 10⁷ 1.5 × 10⁸
Amprenavir (7.66 µg/mL)
— + + +
C
24h
48h
72h
Primary cervix tissue Relative E1^E4 Expression
Amprenavir 7.66 µg/mL
HPV16 (7.5 × 10⁷)
HPV16 (1.5 × 10⁸)
α-V5 Ab (L1)
α-RG1 Ab (L2)
D
24h
48h
72h
Primary cervix tissue Relative E1^E4 Expression
Amprenavir 7.66 µg/mL
HPV16 (7.5 × 10⁷)
HPV16 (1.5 × 10⁸)
α-V5 Ab (L1)
α-RG1 Ab (L2)

E

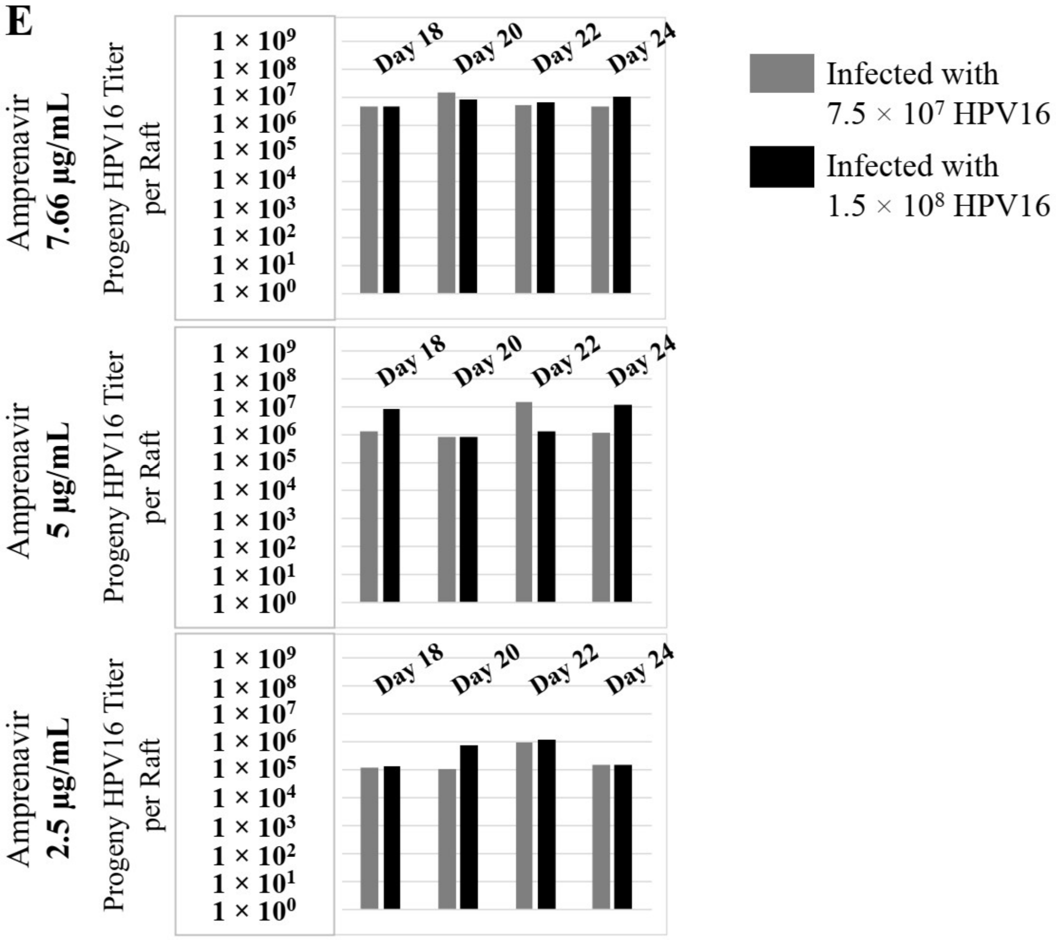

F

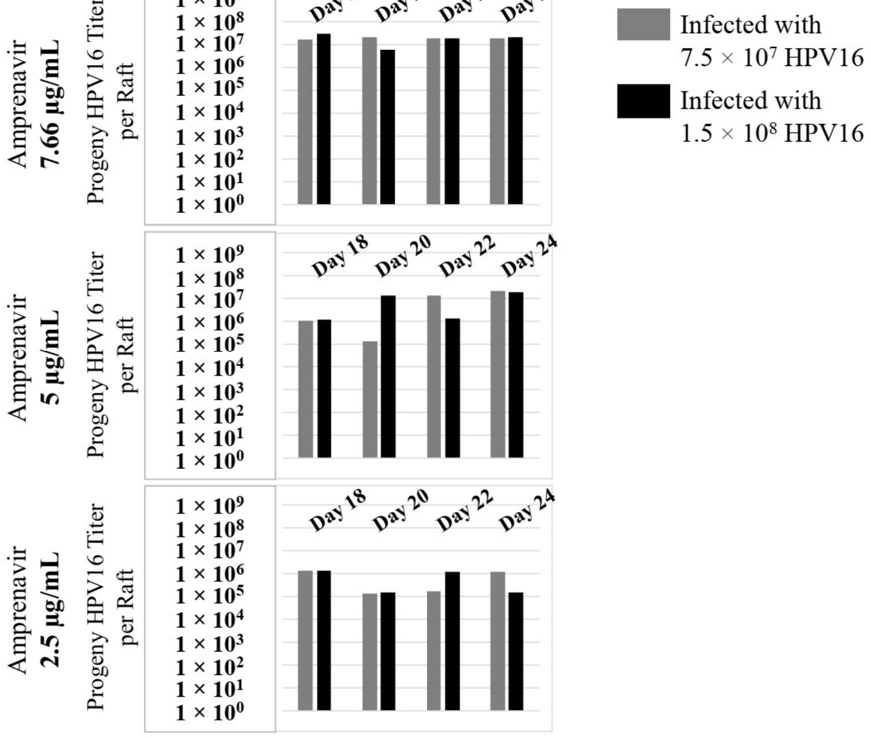

Figure S4. Amprenavir (7.66 µg/mL) Treatment Sensitizes Primary Cervical Tissue to HPV16 Infection. Note: Each panel indicates an individual experiment. (**A,B**) Comparative expression of HPV16 E1^E4 transcripts in Amprenavir treated tissues compared with virus infected tissues not drug treated. (**C,D**) Inhibition of virus infection of Amprenavir treated tissues using HPV16 pre-incubated

with α-V5 and α-RG1. Data was analyzed as mean ± SD. *p*-values were calculated using two-tailed Student's *t*-tests. Significance was based on pairwise Student's *t*-test. Comparisons are indicated as $0.01 < p < 0.05$ by *; $0.001 < p < 0.01$ by **; $0.0001 < p < 0.001$ by ***; and $p < 0.0001$ by ****. (**E,F**) Extended culturing of raft tissues (day 18–24) modulates *prog*-HPV16 titers in an Amprenavir concentration dependent manner.

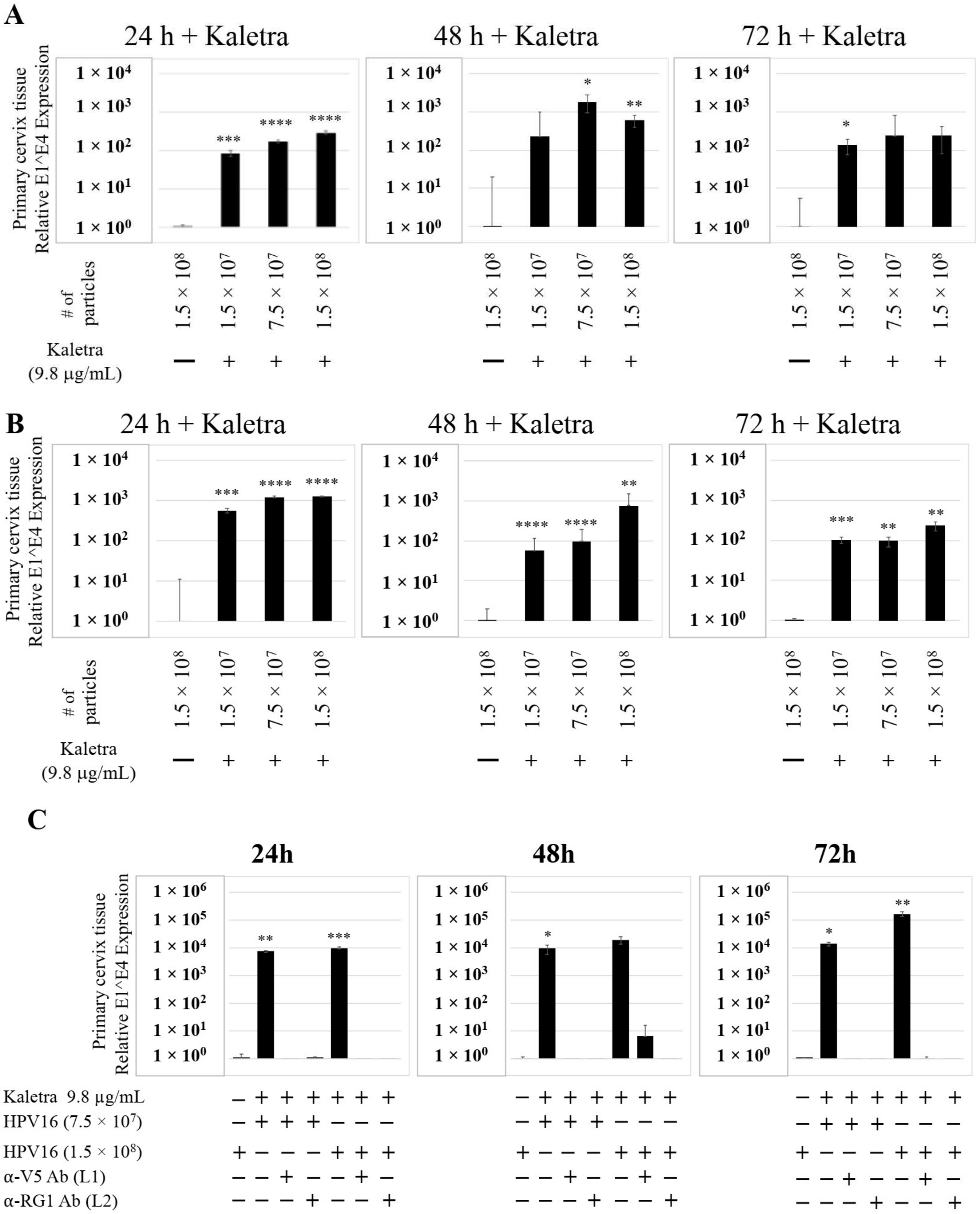

D

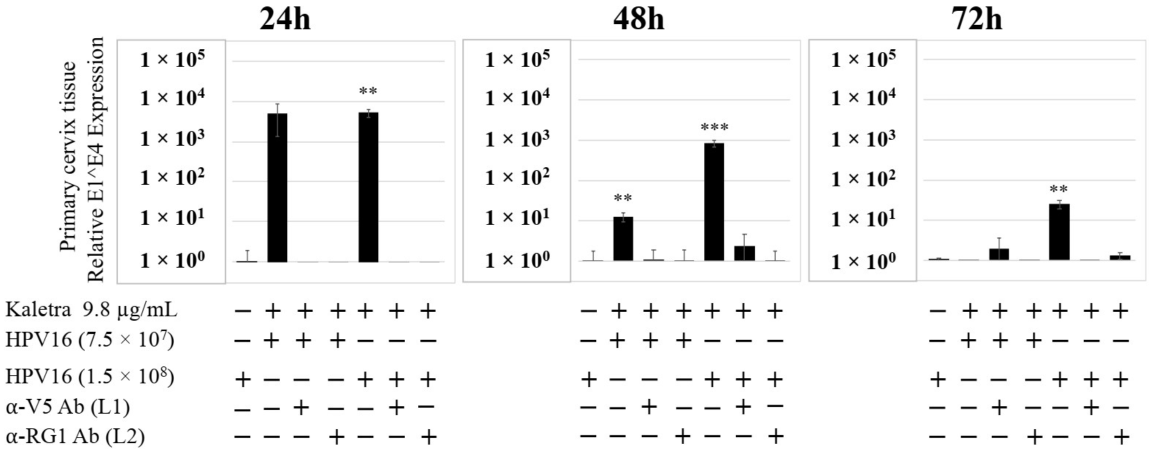
24h
48h
72h
Primary cervix tissue
Relative E1^E4 Expression
1 × 10⁵
1 × 10⁴
1 × 10³
1 × 10²
1 × 10¹
1 × 10⁰
Kaletra 9.8 µg/mL
HPV16 (7.5 × 10⁷)
HPV16 (1.5 × 10⁸)
α-V5 Ab (L1)
α-RG1 Ab (L2)

E

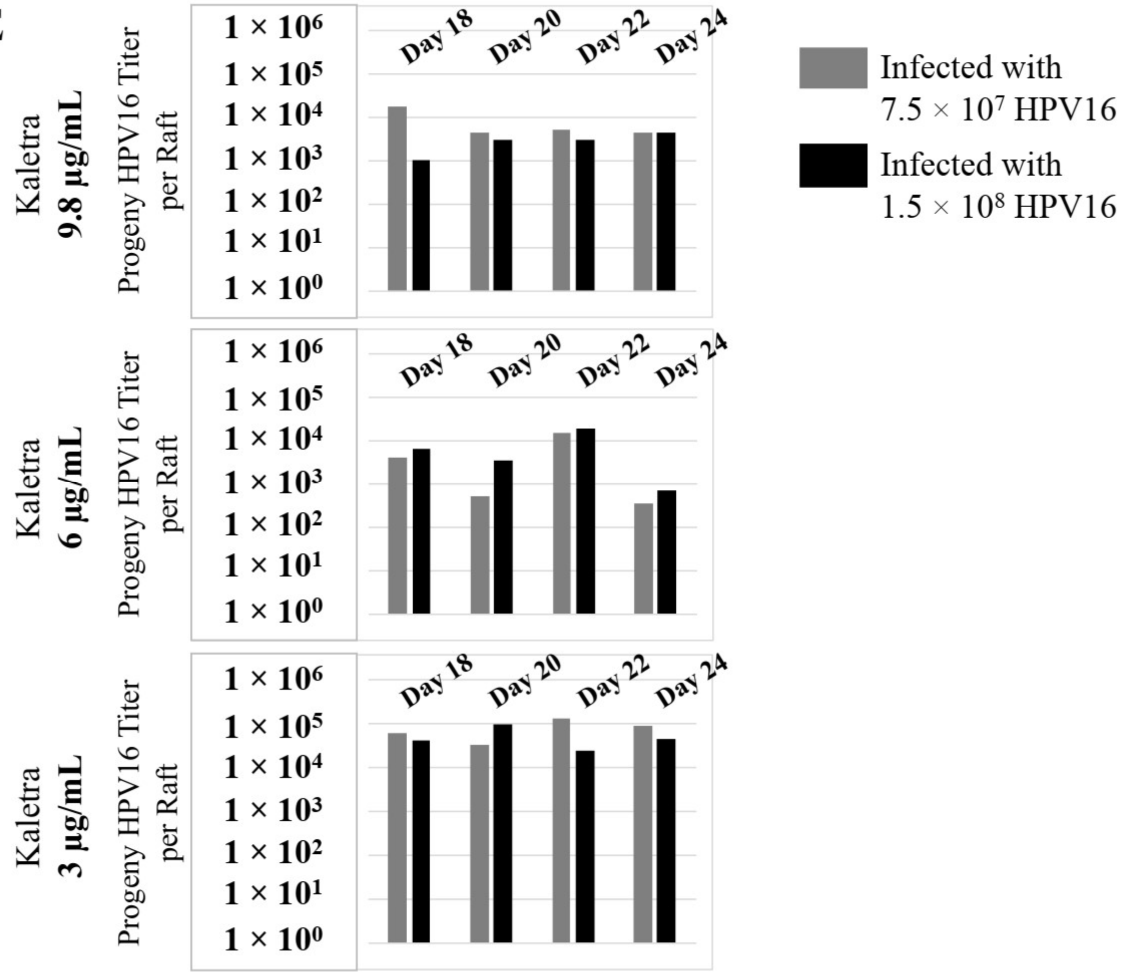
Kaletra 9.8 µg/mL
Kaletra 6 µg/mL
Kaletra 3 µg/mL
Progeny HPV16 Titer per Raft
Day 18
Day 20
Day 22
Day 24
Infected with 7.5 × 10⁷ HPV16
Infected with 1.5 × 10⁸ HPV16

F

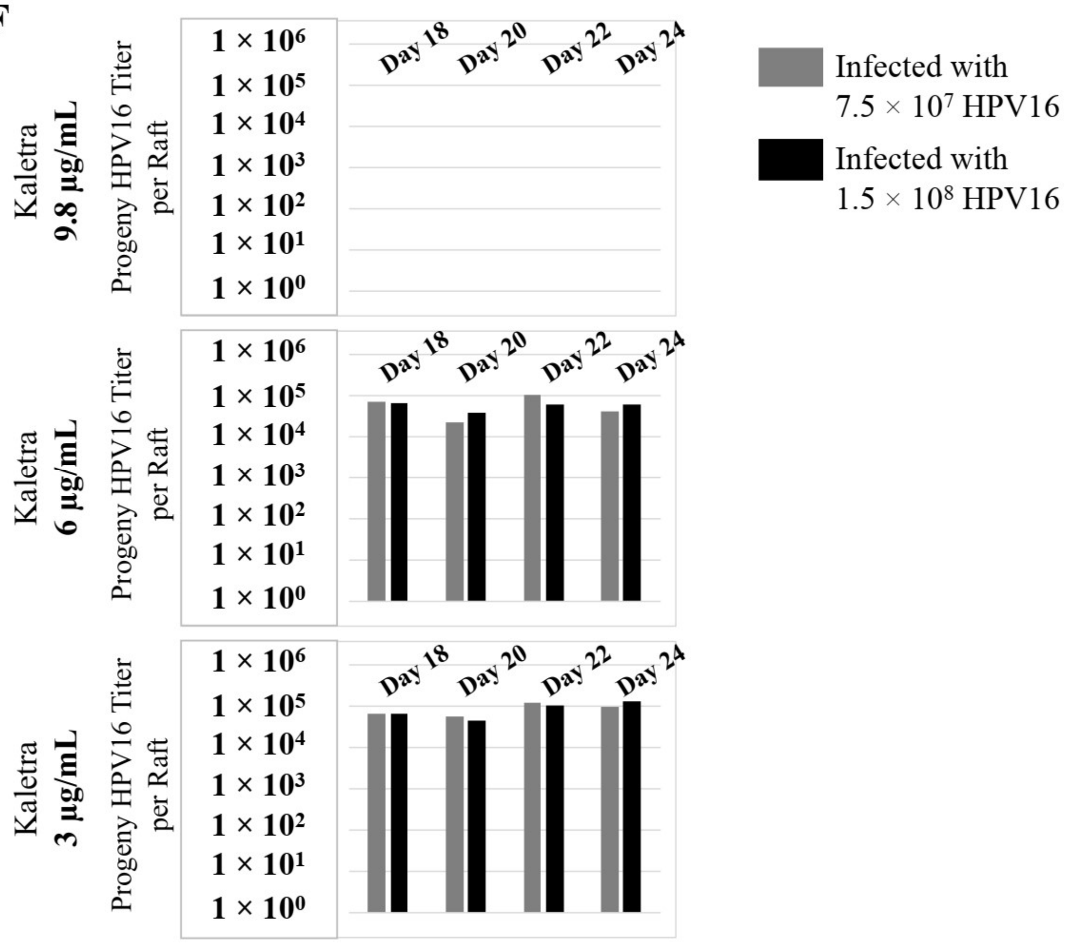

Figure S5. Kaletra (9.8 µg/mL) Treatment Sensitizes Primary Cervical Tissue to HPV16 Infection. Note: Each panel indicates an individual experiment. (**A,B**) Comparative expression of HPV16 E1^E4 transcripts in Kaletra treated tissues compared with virus infected tissues not drug treated. (**C,D**) Inhibition of virus infection of Kaletra treated tissues using HPV16 pre-incubated with α-V5 and α-RG1. Data was analyzed as mean ± SD. p-values were calculated using two-tailed Student's t-tests. Significance was based on pairwise Student's t-test. Comparisons are indicated as $0.01 < p < 0.05$ by *; $0.001 < p < 0.01$ by **; $0.0001 < p < 0.001$ by ***; and $p < 0.0001$ by ****. (**E,F**) Extended culturing of raft tissues (day 18–24) modulates *prog*-HPV16 titers in a Kaletra concentration dependent manner.

Table S1. Primer and Probe Sequences.

HPV16 E2 5′	5′-CCA TAT AGA CTA TTG GAA ACA CAT GCG CC-3′
HPV16 E2 3′	5′-CGT TAG TTG CAG TTC AAT TGC TTG TAA TGC-3′
HPV16 E1^E4 5′	5′-GCT GAT CCT GCA AGC AAC GAA GTA TC-3′
HPV16 E1^E4 3′	5′-TTC TTC GGT GCC CAA GGC-3′
TBP 5′	5′-CAC GGC ACT GAT TTT CAG TTC T-3′
TBP 3′	5′-TTC TTG CTG CCA GTC TGG ACT-3′
Probe HPV16 E1^E4	5′-/56-FAM/CCC GCC GCG ACC CAT ACC AAA GCC/3BHQ-1/-3′
Probe TBP	5′-/5HEX/TGT GCA CAG GAG CCA AGA GTG AAG A/3BHQ-1/-3′

© 2020 by the authors. Licensee MDPI, Basel, Switzerland. This article is an open access article distributed under the terms and conditions of the Creative Commons Attribution (CC BY) license (http://creativecommons.org/licenses/by/4.0/).
